# Amino acid profiles, disease activity, and protein intake in adult patients with Crohn’s disease

**DOI:** 10.3389/fnut.2023.1245574

**Published:** 2023-10-03

**Authors:** Iolanda Cioffi, Olivia Di Vincenzo, Nicola Imperatore, Mariagrazia Fisco, Anna Testa, Filippo Scialò, Fabiana Castiglione, Margherita Ruoppolo, Fabrizio Pasanisi, Lidia Santarpia

**Affiliations:** ^1^Division of Human Nutrition, Department of Food, Environmental and Nutritional Sciences - DEFENS, Università degli Studi di Milano, Milan, Italy; ^2^Department of Clinical Medicine and Surgery, Federico II University Hospital, Naples, Italy; ^3^Department of Public Health, Federico II University Hospital, Naples, Italy; ^4^Gastroenterology and Endoscopy Unit, Santa Maria delle Grazie Hospital, Pozzuoli, Naples, Italy; ^5^CEINGE - Biotecnologie Avanzate F. Salvatore, s.c.ar.l, Napoli, Italy; ^6^Department of Translational Medical Sciences, University of Campania L. Vanvitelli, Naples, Italy; ^7^Department of Molecular Medicine and Medical Biotechnology, University of Naples “Federico II”, Naples, Italy

**Keywords:** glutamine, tryptophan, interleukin-1, inflammatory bowel disease, nutritional status

## Abstract

**Introduction:**

Crohn’s disease (CD) is an immune-mediated inflammatory disorder of the gastrointestinal tract with a relapsing–remitting course. Amino acids (AAs) may play critical roles in the intestinal manifestations of disease, due to their involvement in many metabolic and immune functions. The present study aimed to explore serum AA concentrations in adult patients with CD, looking into their variations due to disease activity, surgery and protein content of diet. Eventually, the link between AAs and inflammatory markers was also assessed.

**Methods:**

Consecutive adult patients aged 18–65 years with diagnosis of CD were recruited. All participants underwent anthropometry and were instructed to fill in a 3-day food record to assess protein intake. Disease activity was clinically defined using the Crohn’s Disease Activity Index (CDAI), while blood samples were taken to analyze serum AA profile and inflammatory markers.

**Results:**

A total of 103 patients with CD (61 men and 42 women; age:39.9 ± 13.9 years, BMI: 23.4 ± 3.51 kg/m^2^) were included. Tryptophan levels were found to be remarkably decreased in most subjects, unrelated to disease activity. On the contrary, concentration of lysine, leucine, valine and glutamine decreased in active versus quiescent CD patients, while aspartic acid, glutamate and glycine increased. The latter AAs were also directly correlated with CDAI and serum interleukin (IL)- 1β concentration. Considering the total protein intake, expressed as g/kg/body weight, we observed a reduction in some essential AAs in patients with unmet protein requirements compared to patients who met the recommendation.

**Discussion:**

In conclusion, specific AAs varied according to disease activity and protein intake, adjusted to body weight and disease status. Glu and Asp concentrations raised with increasing IL-1β. However, extensive research is needed to understand the mechanisms underpinning the link between variation in serum AAs, disease activity and protein intake in patients with CD.

## 1. Introduction

Crohn’s disease (CD) is an immune-mediated inflammatory bowel disease (IBD) characterized by a remitting–relapsing course, able to affect any part of the gastrointestinal tract (1, 2). Chronic inflammation along with malabsorption and/or inadequate dietary intake can facilitate the development of malnutrition in CD patients (3–5), impairing their wellbeing and quality of life (6, 7). So far, studies have shown that some nutrients, such as amino acids (AAs), may have critical functions in the intestinal impact of disease (8–10). AAs are the biosynthetic building blocks of protein macromolecules, and more importantly, they play key roles in cellular metabolism, being involved in the biosynthesis of metabolites with anti-inflammatory and immunoregulatory properties (11, 12). Previous findings have suggested potential therapeutic roles for several AAs (13) in gut-related diseases, particularly for glutamine (Gln), the principal fuel for enterocytes, but current evidence still yields conflicting results (8, 14–17).

From a nutritional point of view, AAs are classified as essential (EAA) or non-essential (NEAA), depending on the body capacity to synthesize *de novo* AAs in adequate amounts to meet optimal requirements (10). However, some NEAAs, such as Gln, glutamate (Glu), arginine (Arg) and glycine (Gly) are defined as conditionally essential, which means that they need to be supplied by the diet when rates of utilization are greater than their synthesis (10, 11). Research on this topic has shown that AA concentrations can vary in serum and tissues with developmental stage, diseases, nutritional state, endocrine status, and physical activity (18).

Variations in AA concentrations have been observed in patients with IBD (11, 19–21) and also in different other conditions such as liver diseases (22, 23), heart failure (24), type 2 diabetes (T2D) (25), obesity (26) and non-small cell lung carcinoma (27). In IBD patients, specific AAs alterations, including reduced levels of Gln, tryptophan (Trp) or histidine (His) have been found in active patients, suggesting that AA profiles might be altered in this population and might reflect disease activity as well as patients’ nutritional status (11, 19, 20, 28).

Another aspect to consider about circulating AAs and their metabolism is dietary habits, since serum AAs, especially EAA concentrations, might be affected by the quantity and quality of habitual protein intake, being diet the key source of EAAs. For instance, it has been previously found in animal studies that serum branched-chain amino acid (BCAA) levels were significantly influenced by cumulative protein intake, indicating that these AAs faithfully reflect the amount of dietary protein intake (29, 30). However, data are at present lacking in humans.

Facing this background, we performed an exploratory research in our cohort of patients with CD (3) aiming to: (1) determine fasting serum levels of EAA and NEAA; (2) verify whether AAs profile might be affected by disease activity, surgery and protein content of diet; and eventually (3) look into associations between serum AAs, protein intake and inflammatory biomarkers.

## 2. Materials and methods

This is a secondary analysis of the cross-sectional REECD (Resting Energy Expenditure evaluation in subjects with Crohn’s Disease) study, where adult patients diagnosed with CD were consecutively recruited from July 2016 to March 2018 at the Department of Clinical Medicine and Surgery, Federico II University Hospital, Naples, Italy. All participants signed informed consent prior to enrolment. The protocol of this study was approved by the Federico II Ethical Committee (No. 102/16) and registered on clinicaltrials.gov as NCT03054935.

As previously reported (3), subjects with a diagnosis of CD and aged between 18 and 65 years were included in this analysis. The exclusion criteria were as follows: use of corticosteroids in the last 3 months; history of acute or chronic liver or kidney disease; current enteral (i.e., tube feeding) or parenteral nutrition, presence of fistulae, ileostomy, or colostomy; presence of extensive small bowel resections (residual small bowel <2 meters); pregnancy or lactation; unstable body weight in the last month; and unable or unwilling to give informed consent.

### 2.1. Clinical data and disease activity

Socio-demographic data, disease duration, previous surgery, smoking habits, drug treatment, location and disease behavior (according to Montreal classification) (31) were collected. Disease activity was clinically defined by the Crohn’s Disease Activity Index (CDAI) (32), classifying patients in the active and quiescent phases (≥ 150 and < 150, respectively).

### 2.2. Anthropometry

Body weight and height were measured to the nearest 0.1 kg and 0.5 cm, respectively, and were taken while the subjects wore light clothes and no shoes using a platform beam scale with a built-in stadiometer (Seca 709; Seca, Hamburg Germany). Body mass index (BMI) was calculated as weight in kilograms divided by the square of the height in meters.

### 2.3. Serum AA analysis and inflammatory markers

AA analysis was performed using patients’ serum samples collected in the morning after a fasting period of 8–10 h. The obtained samples were supplemented with anticoagulant EDTA and stored at −80°C until the subsequent liquid chromatographic (LC) analysis. Aliquots of 500 μL of serum samples were transferred into AMICON-ULTRA filters, adding internal STD, and centrifuged for 10 min at 10,000 rpm in MICROFUGE. Afterwards, approximately 150 μL of filtrate was transferred into 1.5 mL glass screw cap vials with a flat or conical glass bottom insert. Samples were then processed by high-performance liquid chromatography (HPLC) and amino acids contents were measured by an Agilent Technologies 1200 Series LC System using an Agilent Zorbax Eclipse XDB-C18 analytical column (5 μm, 4.6 × 150 mm) and Agilent Eclipse XDB-C18 analytical guard column (5 μm, 4.6 × 12.5 mm). Metabolites derivatization was performed in automated mode using ophthalaldehyde (OPA) and 9-fluorenylmethyl chloroformate (FMOC) for primary and secondary amino acids, respectively. The chromatographic separation was carried out using 40 mM phosphate buffer pH 7.8 as solvent A and CH_3_CN/CH_3_OH/H_2_O (40/40/20) as solvent B. The flow rate was set at 1.3 mL/min and temperature at 40°C. The linear gradient was the following: from 10 to 20% of solvent B in 6 min, from 20 to 27% of solvent B in 6 min, from 27 to 60% of solvent B in 10 min, from 60 to 100% of solvent B in 2 min plus an isocratic step to 100% of solvent B during 6 min. The single amino acids were identified according to their retention time and quantified to compare absorption in respect to standard compounds in the calibration solution, a mixture 200 μM of amino acids.

Total EAAs were calculated as the sum of valine (Val), isoleucine (Ile), leucine (Leu), methionine (Met), lysine (Lys), phenylalanine (Phe), Trp, threonine (Thr), and His; while BCAAs were calculated as the sum of Val, Ile, and Leu. Total NEAAs were calculated as the sum of Arg, Gly, alanine (Ala), serine (Ser), tyrosine (Tyr), cystine (Cys), asparagine (Asn), Gln, aspartic acid (Asp), and Glu. The non-proteinogenic AAs like ornithine (Orn), citrulline (Cit), and taurine (Tau) were also evaluated, due to their role in human protein metabolism.

Serum tumor necrosis factor (TNF)-α, interleukin (IL)-1β, and IL-6 were analyzed to assess the inflammatory status. They were examined by automated microfluidic immunoassay cartridges on ProteinSimple Ella (Bio-Techne^®^ Minneapolis, United States), in accordance with the manufacturer’s instructions. While, C-reactive protein (CRP) (mg/L) was assessed at the centralized laboratory of Federico II University Hospital following standardized techniques, as previously stated (3, 33).

### 2.4. Protein content of diet

Total intake of protein was retrieved from 3-day food diary. All diaries were calculated using the WINFOOD database (3.4 version; Medimatica, Teramo, Italy). As described elsewhere (4), patients were carefully instructed by a registered dietitian to fill in a food diary for 3 non-consecutive days (2 weekdays and 1 weekend day) before coming to the hospital. A dedicated dietitian reviewed the completed 3-day food diary upon return for clarification of portions, missing or unclear data, and food preparation methods.

To assess the effects of protein amount on serum AA concentrations, we used as reference values the protein requirements of about 1.0 g in remission and 1.2 g in active phase, suggested by the ESPEN guideline in adults with IBD (34), to split our sample. Data are expressed as gram of protein for each kilogram of body weight (g/kg/body weight).

### 2.5. Statistical analysis

Data were expressed either as the means and standard deviation (SD) or as medians and interquartile range (IQR) or N (%), depending on the type and distribution of data. The percentage of CD patients whose serum AAs concentration was below, within or above the reference range, provided by the laboratory, was calculated for each AA. The Kolmogorov–Smirnov Test and the Shapiro–Wilk Test were used as tests of normality to examine whether variables were normally distributed. Differences in AAs concentration between active and quiescent CD groups were compared by unpaired t-tests for parametric variables, while the Mann–Whitney U test was applied for nonparametric data. The chi-squared test was used for differences between categorical variables. Spearman correlations analysis was used to assess the relationship between individual AA level, protein intake and inflammatory markers. A *p-*value <0.05 was considered statistically significant. Statistical analyses were conducted using the SPSS Statistics software (version 28.0.0, SPSS Inc., Chicago, IL, United States).

## 3. Results

One hundred and forty-eight patients with CD participated in the study. A total of 45 were ruled out from this secondary analysis because 8 subjects did not meet the inclusion criteria and 37 lacked the additional sample for analyzing AA profile. Therefore, 103 patients, including 62 men and 41 women, were selected, of which 55 patients were in the quiescent phase (CDAI<150) and 48 showed mild to moderate activity (150 > CDAI <450).

The socio-demographic and clinical characteristics of patients, divided by sex, are summarized in Table 1. The study group showed a mean age of 39.9 ± 13.9 years with an average BMI of 23.4 ± 3.51 kg/m^2^, where mostly had a normal BMI (72%) and were non-smokers (61%). Based on the Montreal classification, disease was mainly diagnosed between 17 and 40 years (68%), located in the ileum-colon (52%) and characterized by a stricturing phenotype (52%). Regarding pharmacological therapies, 40% of patients were treated with biologic agents (adalimumab, infliximab and vedolizumab) whereas almost 30% were not being treated at that time mostly due to the screening phase before starting biologic therapy.

**Table 1:** Demographic and clinical characteristics of CD patients.

	Total	Men	Women
N, (%)	103 (100)	62 (60.2)	41 (39.8)
Age (years), mean [SD]	39.9 [13.9]	38.7 [13.4]	41.6 [14.5]
Body weight (kg), mean [SD]	65.5 [12]	70.6 [10.5]	57.8 [9.58]
BMI, n (%)			
<18.5 kg/m^2^	6 (5.8)	1 (1.6)	5 (12.2)
18.5–24.9 kg/m^2^	74 (71.8)	48 (77.4)	26 (63.4)
25–29.9 kg/m^2^	18 (17.5)	10 (16.1)	8 (19.5)
>30 kg/m^2^	5 (4.9)	3 (4.8)	2 (4.9)
Previous surgery, n (%)	54 (52.4)	29 (46.8)	25 (61)
Disease duration (years), median [range]	6.50 [1–30]	6.42 [1–30]	6.58 [1–23]
Currently smoking habits n (%)			
Yes	22 (21.4)	11 (17.7)	11 (26.8)
No	63 (61.2)	40 (64.5)	23 (56.1)
Ex-smoker	18 (17.5)	11 (17.7)	7 (17.1)
Clinical activity, n (%)			
CDAI <150	55 (53.4)	36 (58.1)	19 (46.3)
>150 CDAI <450	48 (46.6)	26 (19.4)	22 (53.7)
Montreal age at diagnosis, n (%)			
A1: < 16 y	18 (17.5)	11 (17.7)	7 (17.1)
A2: 17–40 y	70 (68.0)	43 (69.4)	27 (65.9)
A3: > 40 y	15 (14.6)	8 (12.9)	7 (17.1)
Montreal disease location, n (%)			
L1: Ileum	36 (35)	20 (32.2)	16 (39.0)
L2: Colon	10 (9.7)	10 (16.1)	0
L3: Ileum and colon	54 (52.4)	30 (48.4)	24 (58.5)
L4: Upper GI tract	3 (2.9)	2 (3.2)	1 (2.4)
Montreal disease behavior, n (%)			
B1: Inflammatory	32 (31.1)	24 (38.7)	8 (19.5)
B2: Stricturing	54 (52.4)	32 (51.6)	22 (53.7)
B3: Penetrating	17 (16.5)	6 (9.7)	11 (26.8)
Perianal disease, n (%)	19 (18.4)	12 (19.4)	7 (17.1)
Medications, n (%)			
None	30 (29.1)	19 (30.6)	11 (26.8)
5-ASA	17 (16.5)	11 (17.7)	6 (14.6)
IMMs	15 (14.6)	8 (12.9)	7 (17.1)
Biologics	41 (39.8)	24 (38.7)	17 (41.5)

A, Age at diagnosis; ASA, Amino Salicylic Acid; B, disease Behavior; BMI, Body Mass Index; CDAI, Chron’s Disease Activity Index; IMMs, Immunosuppressives; L, disease Location; SD, Standard Deviation; y, year.

**Figure 1:**
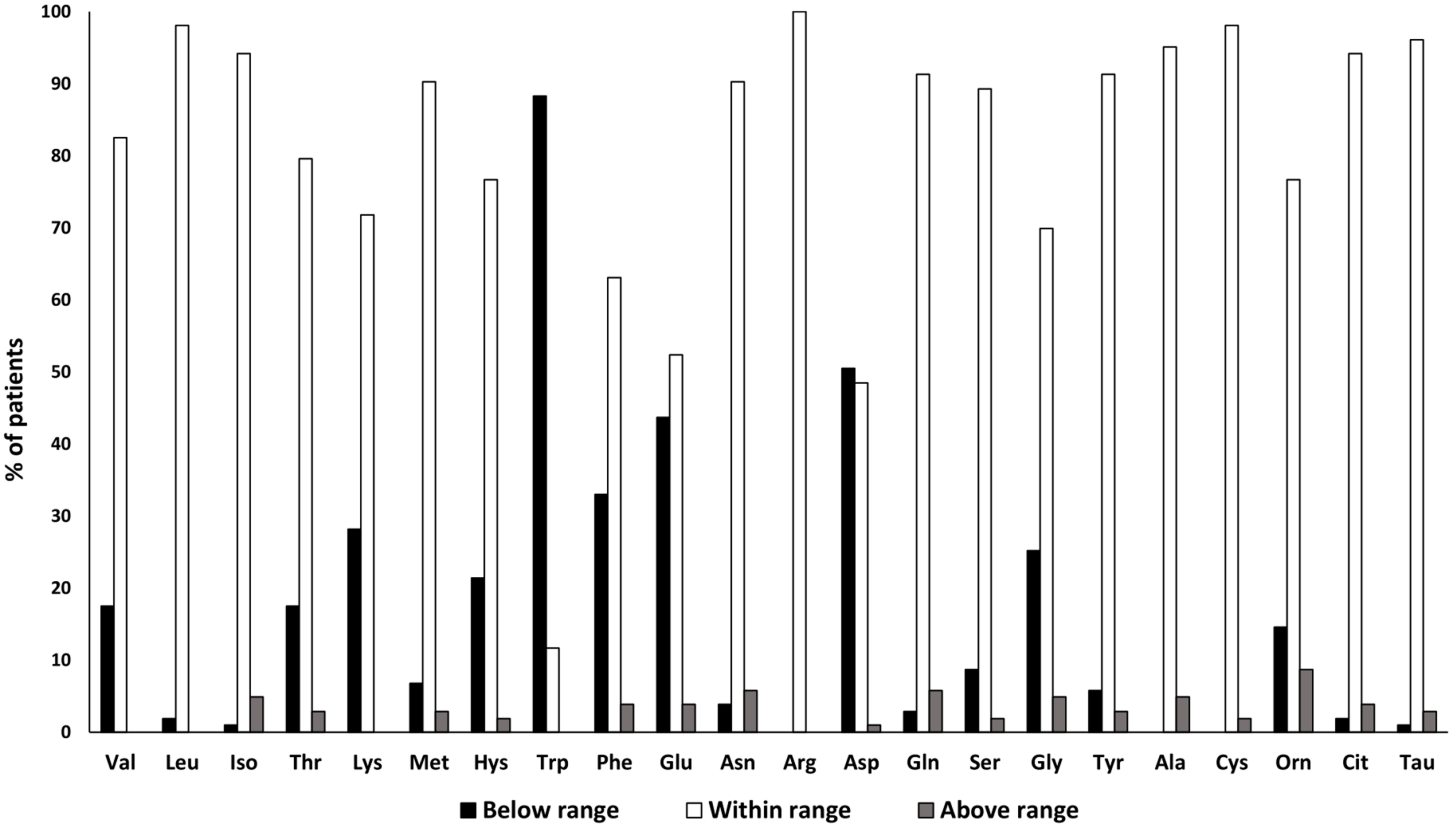
Data are expressed as percentage of patients resulting below (black column), above (gray column) or within (white column) the reference range considered by the laboratory.

Based on the reference ranges (35, 36), the percentage of patients showing serum AAs content below, within and above range limits was calculated. Results indicated that most patients had a serum AAs content within the reference range, except for Trp, Asp, and Glu levels. In detail, Trp levels were below the reference range in 90% of patients; conversely, both Asp and Glu levels were lower than the reference range in 50% of them (Figure 1).

### 3.1. Differences in serum AAs according to disease activity

**Figure 2:**
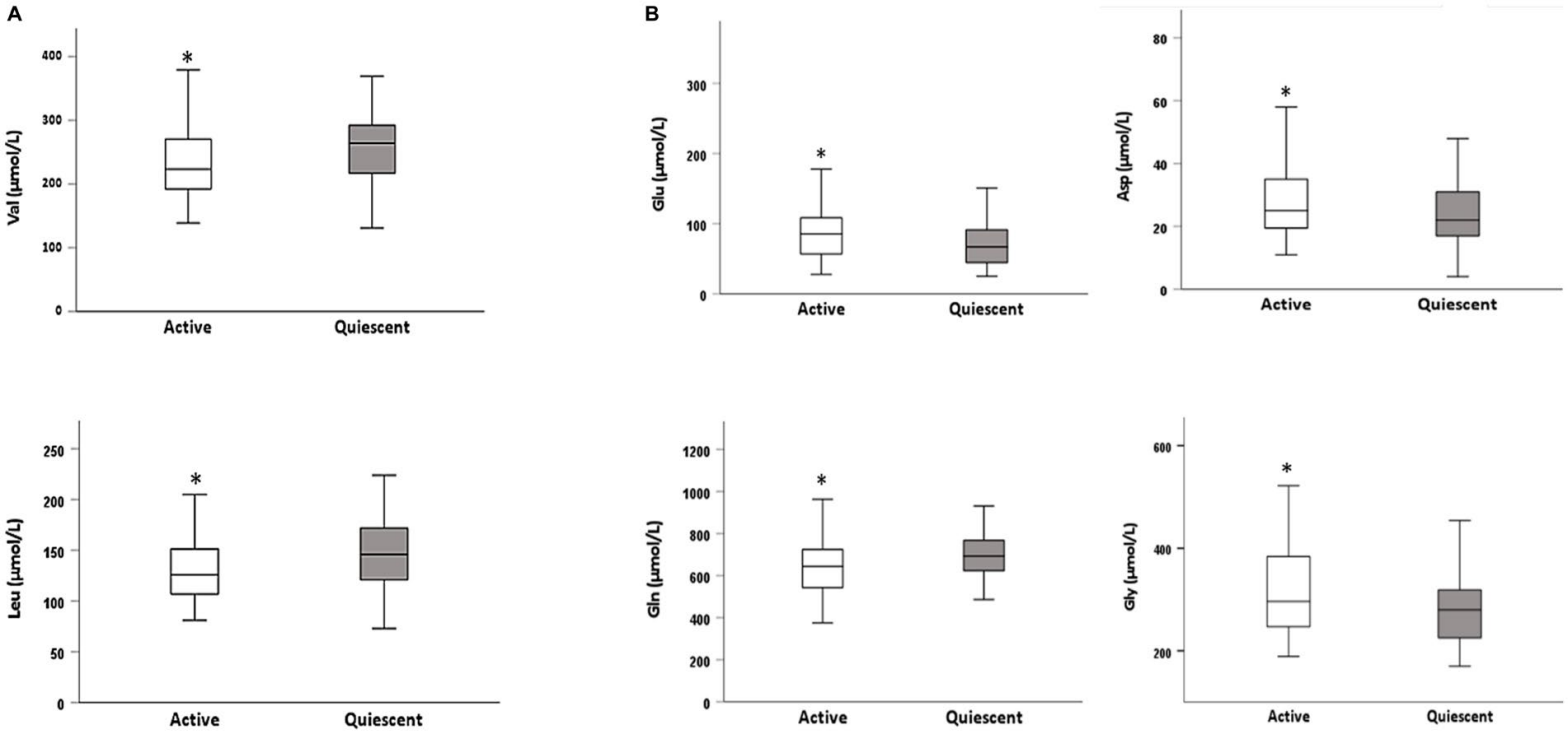
Differences in AA concentrations between active and quiescent CD patients: **(A)** essential amino acids (EAAs) and **(B)** non-essential amino acids (NEAA). Differences between valine (Val), leucine (Leu), glutamate (Glu), aspartic acid (Asp), glutamine (Gln), and glycine (Gly) are presented as box plots, reporting median, first and third quartile, minim and maximum. Stars indicate significant differences as determined by the Mann–Whitney U test. *p < 0.05.

AA concentrations detected in the whole sample as well as in clinically active and quiescent CD patients are summarized in Table 2. Data are presented as EAAs, NEAAs, and other metabolites, the latter including Orn, Cit, and Tau. Findings revealed lower concentrations of serum Val, Leu and Gln in active CD patients compared to those clinically quiescent, but increased levels of Glu, Asp, and Gly, as presented in Figure 2. An overall tendency toward a reduction of BCAAs was found in active patients versus those clinically quiescent, albeit not statistically significant (*p* = 0.07). AA levels did not differ according to disease location and behavior, except for Cys concentration which resulted significantly reduced in patients with penetrating behavior (B3) compared to those with stricturing phenotype (B2), unrelated to disease activity [B3:27.6 (2.37) μmol/L vs. B2: 34.1 (1.30) μmol/L; *p* = 0.03].

**Table 2:** Serum amino acids concentration in patients with CD according to disease activity.

	All	Active	Quiescent	Reference (35, 36)
EAA, μM/L				
Valine	233 (79)	223.5 (84)*	264 (76)	*187–411*
Leucine	139 (57)	127 (59.3)*	146 (53)	*82–258*
Isoleucine	74 (26)	73.5 (26)	74 (25)	*42–111*
Threonine	146 (65)	147.5 (64.3)	145 (67)	*109–268*
Lysine	176 (60)	169.5 (51)	183 (66)	*150–286*
Methionine	28 (10)	26.5 (10)	29 (10)	*21–44*
Histidine	88 (22)	84 (24.8)	90 (18)	*72–131*
Tryptophan	6 (3)	6 (3)	7 (3)	*10–30*
Phenylalanine	64 (17)	60 (17.3)	67 (14)	*59–112*
BCAA	445 (149)	421 (156)	484 (154)	*–*
Total EAAs	908 (264)	862 (253)	957 (263)	*–*
NEAA, μM/L				
Glutamic acid	73 (45)	85.5 (52.3)*	67 (50)	*67–223*
Asparagine	60 (15)	59.9 (15.8)	60 (16)	*43–87*
Arginine	101 (34)	95.5 (37)	107 (28)	*36–172*
Aspartic acid	23 (16)	25 (15.8)*	22 (14)	*24–102*
Glutamine	664 (141)	644 (187.5)**	693 (42)	*432–871*
Serine	147 (42)	147.5 (37.3)	146 (42)	*111–297*
Glycine	287 (113)	296.5 (139)*	280 (98)	*240–460*
Tyrosine	62 (20)	61 (21.8)	62 (17)	*45–107*
Alanine	379 (132)	376.5 (143.5)	397 (129)	*193–597*
Cysteine	31 (12)	30.5 (12.5)	31 (11)	*5–55*
Total NEAAs	1864 (417)	1853 (469)	1890 (316)	*–*
Other metabolites, μM/L				
Ornithine	93 (12)	99.5 (46.8)	91 (44)	*63–133*
Citrulline	33 (11)	33 (10.8)	33 (14)	*15–57*
Taurine	151 (70)	150 (57)	155 (78)	*50–270*

Data are expressed as median and interquartile range. BCAA, branched-chain amino acid; EAAs, essential amino acids; NEAAs, non-essential amino acids. **p < 0.01; *p < 0.05.

### 3.2. Differences in serum AAs according to previous surgery

Since half of patients had surgery due to medically refractory disease or disease complications, as shown in Table 1, we examined whether serum AA concentration was affected by previous surgery. Overall, AA levels seemed not to be affected by surgery (Supplementary Table S1), although a trend was observed in Val and Asp concentrations. Specifically, decreased Val and increased Asp levels were found in patients undergone surgery compared to those who did not [Val: 223 (84.5) μmol/L vs. 255 (80.5) μmol/L, *p* = 0.07; Asp: 26.5 (16.5) μmol/L vs. 23 (12.5) μmol/L, *p* = 0.06, respectively].

Eventually, we also looked into the effect of surgical site on AA concentrations. Considering that surgical patients had the following resections: ileum (31.5%), colon (37%), and ileum-colon (31.5%), the concentration of serum AAs did not differ among the three groups.

### 3.3. Differences in serum AAs according to total intake of protein and disease activity

Protein content of diet retrieved from 3-day food records was 71.2 ± 22.5 g, differing between men and women (M: 79.4 ± 23.6 g vs. W: 60.4 ± 14.7 g; *p* = 0.001) and representing about 17% of total energy intake. The daily amount of protein was expressed as g/kg of body weight, observing an overall consumption of 1.11 ± 0.36 g/kg/body weight in the whole sample. However, by applying the protein requirements based on disease activity status, i.e., 1.0 g/kg body weight in remission and 1.2 g/kg body weight in active phase, as suggested by the last ESPEN guideline in IBD patients (34), we found that 47 (46%) out 103 patients did not meet the protein requirements.

Therefore, we investigated whether differences in daily protein intake adjusted by body weight could have had an impact on serum AA concentrations, in particular on EAAs. To simplify, the group of patients with an intake below the protein requirements was called “unmet protein intake (UPI) group” and the one within or above the protein requirements was referred to “met protein intake (MPI) group.” Findings revealed differences in serum EAA levels between the two groups, since concentrations of Thr, Met, Lys and Phe resulted reduced in the UPI group; whereas no variation was observed for NEAAs concentrations, apart from Arg levels which were lower in the UPI than in the MPI group, as presented in Table 3.

**Table 3:** Serum amino acids concentration according to protein requirements in patients with CD.

	UPI *N* = 47	MPI *N* = 56
EAA, μM/L		
Valine	224 (91)	259 (76)
Leucine	131 (48)	140 (55)
Isoleucine	72 (26)	77 (29)
Threonine	134 (61)*	156 (54)
Lysine	170 (41)**	190 (68)
Methionine	26 (8)*	29 (10)
Histidine	88 (23)	89 (20)
Tryptophan	6 (2.1)	7 (4)
Phenylalanine	61 (15)*	66 (20)
BCAA	424 (157)	484 (144)
Total EAAs	873 (231)	948 (246)
NEAA, μM/L		
Glutamic acid	74 (42)	70.5 (47)
Asparagine	59 (15)	60 (18)
Arginine	96 (43)*	108 (38)
Aspartic acid	23 (14)	24.5 (17)
Glutamine	684 (172)	662 (130)
Serine	145 (42)	149 (40)
Glycine	280 (151)	291 (73)
Tyrosine	61 (20)	62.5 (20)
Alanine	399 (104)	367 (134)
Cysteine	32 (13)	29 (10)
Total NEAAs	1892 (507)	1846 (288)
Other metabolites, μM/L		
Ornithine	92 (44)	93.5 (42)
Citrulline	34 (11)	33 (14)
Taurine	149 (71)	154.5 (72)

BCAA, branched-chain amino acid; EAAs, essential amino acids; IQR, interquartile range; MPI, Met protein intake; NEAAs, non-essential amino acids; UPI, Unmet protein intake. **p < 0.01; *p < 0.05.

Moreover, we performed the analysis between UPI and MPI group by stratifying to disease activity (Supplementary Table S2). In active patients, findings showed that both Thr and Arg levels were lower in the UPI group compared to the MPI group [UPI: 134 (69) μM/L vs. MPI:164 (31) μM/L, *p* = 0.054; UPI: 89 (45) μM/L vs. MPI: 101 (40) μM/L, *p* = 0.032, respectively]. While Lys was the only AA that differed in the quiescent group [UPI: 178 (57) μM/L vs. MPI: 195 (55) μM/L, *p* = 0.024].

### 3.4. Relationship between AAs, disease activity and inflammatory markers

**Figure 3:**
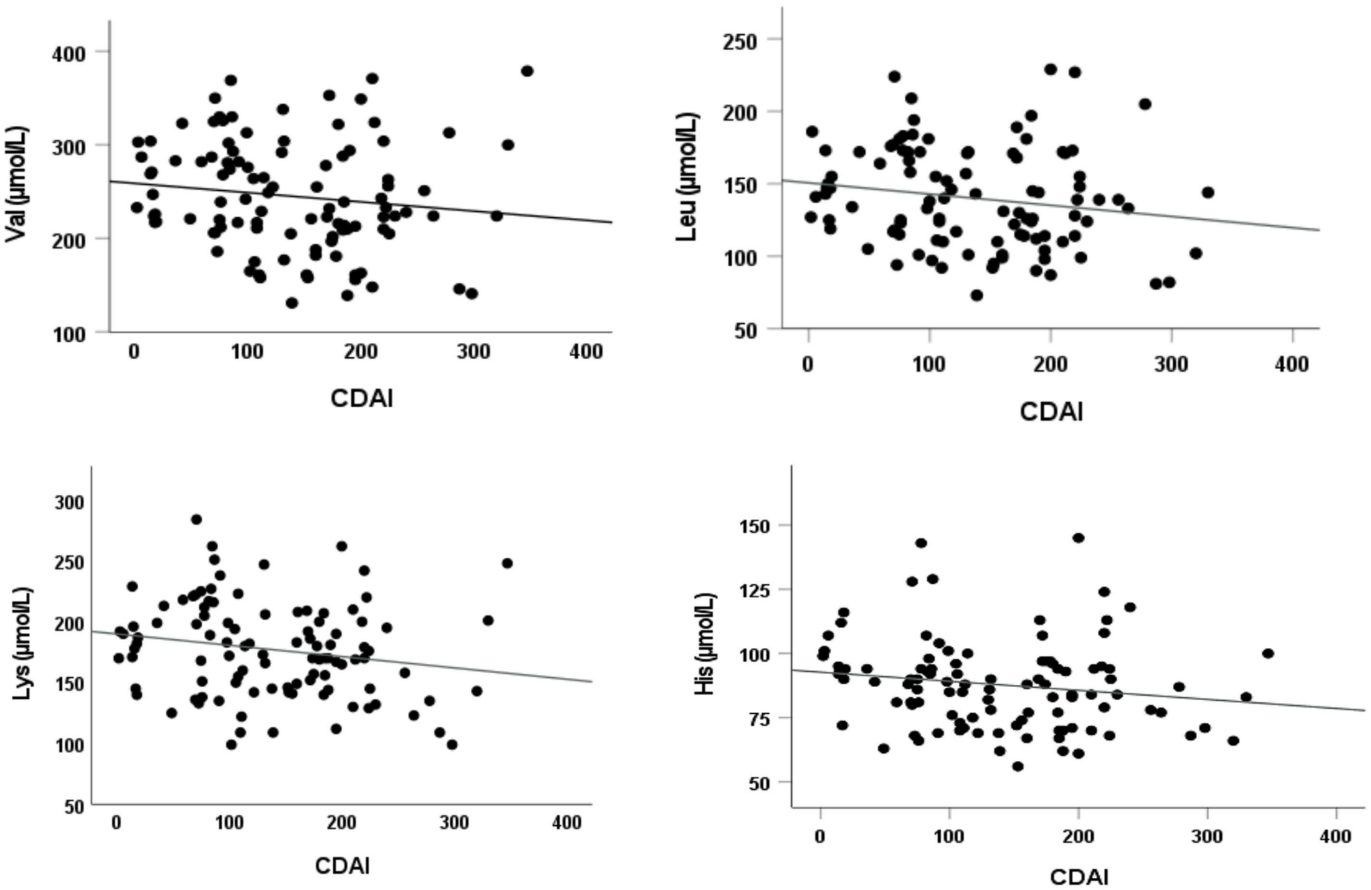
Correlations between serum essential AA levels and disease activity. Significant inverse Spearman’s rank correlation coefficients between the following essential AAs valine (Val), leucine (Leu), lysine (Lys), and histidine (His) and disease activity (CDAI).

Finally, the relationships between serum AA concentrations, CDAI, CRP and cytokines were investigated. Data showed that among EAAs, Val (*r* = −0.190; *p* = 0.05), Leu (*r* = −0.208; *p* = 0.04), Lys (*r* = −0.223; *p* = 0.02), and His (*r* = −0.190; *p* = 0.05) were inversely associated with CDAI (Figure 3).

**Figure 4:**
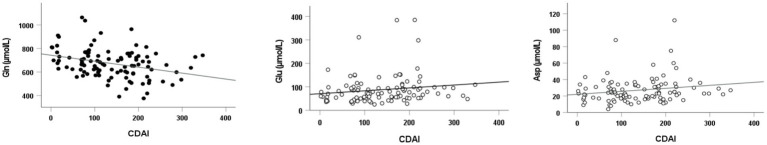
Correlations between serum non-essential AA levels and disease activity. Significant Spearman’s rank correlation coefficients between the following non-essential AAs: glutamine (Gln) glutamate (Glu), and aspartic acid (Asp) and disease activity (CDAI).

With regards to NEAAs, Gln (*r* = −0.314; *p* = 0.001) was negatively correlated to disease activity, whereas both Glu (*r* = 0.216; *p* = 0.03), and Asp (*r* = 0.193; *p* = 0.05) displayed a positive correlation (Figure 4). None of the above AAs was significantly correlated to CRP.

**Figure 5:**
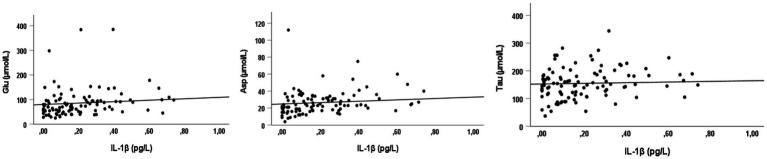
Correlations between Glu, Asp, Tau levels and IL-1β. Significant direct Spearman’s rank correlation coefficients between glutamate (Glu), aspartic acid (Asp), and taurine (Tau) and a proinflammatory cytokine (interleukin-1beta).

Interestingly, Glu (*r* = 0.357; *p* = 0.001), Asp (*r* = 0.423; *p* = 0.001), and Tau (*r* = 0.250; *p* = 0.011) levels were found to be directly associated to IL-1β concentrations, as reported in Figure 5, while no correlations were observed for IL-6 and TNF-α.

## 4. Discussion

So far, accumulating evidence has highlighted the role of AAs in the maintenance of intestinal and immune homeostasis in IBD (9, 35). AAs derived from both endogenous and exogenous proteins (i.e., diet) and their availability is essential in many key processes of metabolic and immune responses (12, 37). Some AAs, such as Trp or Cys, are metabolized by gut bacteria in the colon to synthesize proteins and other crucial components (38–40). Because of their contribution toward keeping integrity/functionality of the intestine by modulating gut inflammation, specific AAs, like Gln, have been tested in several pre-clinical IBD studies as therapeutic agents, with promising results (10, 41). However, clinical trials have failed to demonstrate any beneficial effects of AAs supplementation in patients with CD (8, 14), requiring further research.

This is an exploratory analysis aimed to assess serum AA profiles in a cohort of patients with CD, considering the effects of disease activity, surgery and protein intake. In addition, the link between AAs, disease activity and inflammatory markers was evaluated. Briefly, our findings highlighted that Lys, Leu, Val and Gln decreased in active CD patients compared to those clinically quiescent, and together with His were inversely correlated to CDAI. While Asp and Glu increased in active patients, resulting in a direct association with CDAI and IL-1β. Only Trp levels were found to be remarkably decreased, unrelated to CDAI. Finally, we observed a reduction in some EAAs between patients with different protein requirements.

To date, only a few clinical studies have examined serum AA profiles in patients with IBD, taken alone (11, 20) or in comparison with controls (19, 21, 39), setting out how the variation of AAs might be linked to disease severity, with different results. Hisamatsu et al. (19) showed reduced levels of His and Trp in active subjects compared to quiescent CD patients, reporting an inverse correlation of His, Glu, Met, Asn, Trp, and Ala with CDAI and serum CRP level (*p* < 0.01). While Chiba et al. (11) did not find any differences in AAs levels between active and inactive CD patients (probably given the small sample size), but they observed that Val, Met, Leu, His, and Trp levels were inversely correlated with CDAI, as we found. Not surprisingly, when data from IBD patients were compared to those of controls, differences in AAs levels were even more pronounced (19, 21, 28), confirming that metabolic disturbances might be a characteristic of IBD patients (42).

Opposite trends in AA concentrations among patients with a different inflammatory status might be explained by the interconnection of their metabolisms and functions, like between Gln and Glu (43, 44). Gln is the richest AA in the body and is involved in many physiological and metabolic functions, acting not only as a key precursor of nucleic acids and nucleotides, but also as a relevant provider of Glu (17, 45) and vice versa (46). Current findings suggest that Gln has a key role in improving intestinal permeability, preventing mucosal atrophy, and maintaining gut integrity (43). Moreover, it has been reported that Gln may have an antioxidant and anti-inflammatory activity in patients with IBD (47), by reducing the production of several proinflammatory cytokines (IL-8 and IL-6) and enhancing the production of the anti-inflammatory IL-10 in patients with CD (9).

On the other hand, Glu is the major excitatory neurotransmitter in the central nervous system (CNS) and in the periphery, including the enteric nervous system (ENS) (47, 48). It is also a precursor of different neuroactive molecules produced within the intestinal mucosa such as Orn, Arg and glutathione, with anti-oxidant activities (49). As such, Glu may play a prominent role in the transduction of local inflammatory signals (50), being one of the neuromodulators/ neurotransmitters involved in the regulation of the microbiota-gut-brain axis (51). Thus, it is likely that the relationship among increased levels of Glu, decreased Gln concentrations and disease activity in CD patients might be related to the poor ability of the intestinal mucosa to resist oxidative stress, increasing the inflammatory response (42). In fact, Glu has been found to be directly associated with serum levels of IL-1β, a pro-inflammatory cytokine. To our knowledge, this is the first clinical study assessing the link between serum AAs and cytokines in patients with CD. For example, in many neuro-degenerative diseases, typically characterized by an elevated inflammation state, the increase in both intra- and extracellular Glu might be induced by high levels of pro-inflammatory cytokines, especially IL-1β and TNF-α (51). Unfortunately, we were not able to demonstrate the link between Glu and IL-1β in CD, especially in the active phase, but this may be worth future investigations.

Unless other AAs, Trp levels were reduced in all CD patients, unrelated to CDAI, in line with previous data from literature (39). Actually, low Trp levels have been reported in patients with various autoimmune disorders, including IBD (19, 52, 53). Quite recently, a systematic analysis showed that serum Trp levels were lower in IBD patients than in healthy controls (39). Trp is an essential AA involved in numerous physiological functions like intestinal intracellular protein turnover, microbiota diversity, and in reducing intestinal inflammation (43). In addition, being Trp a precursor of serotonin biosynthesis, reduced Trp concentrations could determine poor quality of life and depression, while Trp deficiency may result in a persistent immune activation (54). Also, a reduced availability of serum Trp might not strictly depend on dietary intake, since a previous study (39) reported that dietary intake of Trp was not significantly different between active and inactive patients, as we found (Table 3).

Since a significant part of AAs and oligopeptides are absorbed mainly in the proximal jejunum (10, 18, 55), it is plausible that alterations of the gastrointestinal tract like intestinal resections or disease behavior could potentially affect the serum concentrations (10). Our data showed that AA levels did not differ according to surgery (yes/no) as well as to the surgical sites (ileum/colon/ileum-colon). But it is important to highlight that none of patients received jejunal resections and all had at least 2 meters remnant bowel, sufficient to guarantee a satisfactory nutrient absorption. Indeed, looking at Cit levels, a non-proteinogenic AAs mainly produced by enterocytes and proposed as quantitative biomarker of their mass and function in different diseases (56), they were similar between the above-mentioned groups. With regard to disease behavior, classified by the Montreal classification (31), differences in Cys concentration, a sulfur-containing AA with strong antioxidant activities and precursor of glutathione (13), were found between patients with penetrating (B3) and stricturing behavior (B2). Although this finding might be interesting given the role of Cys in the regulation of cellular redox state (43), it should be outlined that the group of patients classified as B3 (16%) was smaller in comparison with the B2 (52%) (Table 1), potentially affecting the results.

On the other hand, Tau, another non-proteogenic AA, derived from Cys metabolism, was found to be positively associated to IL-1β concentration. Tau is involved in many physiological processes such as osmoregulation, anti-oxidative responses and membrane stabilization, playing major roles in both anti-inflammatory and antioxidant responses (9, 13). A recent study analyzing differences in serum AAs between IBD patients reported that significantly elevated levels of Tau were a distinctive feature of CD (42).

Last, but not least, we looked into the possible role of protein intake on the concentrations of serum AAs. Most AAs are metabolized during the journey from the intestinal epithelium to the portal bloodstream and it is likely that their circulating levels might not be indicative of the intake with the diet (55). Animal studies showed that chronic alteration of protein intake affects fasting concentrations (29), while data in humans are currently unavailable. Our findings showed some variations in serum Thr, Met, Lys, Phe, and Arg levels between patients meeting and those not meeting protein requirements provided by the ESPEN guideline (34). Additionally, similar results were substantially observed when subjects were stratified to disease activity. Indeed, the quantity of ingested protein, adjusted by body weight and disease activity, is not the only variable to consider for meeting protein requirements, since quality of AAs is extremely relevant in the entire process (55). In addition, it has been widely demonstrated that nutritional deficiencies observed in patients with CD are the result not only of insufficient dietary intake, but also of malabsorption and complications relative to the metabolic disturbances induced by the disease.

The present study has several limitations. Since the measurement of fasting AA concentrations might not be indicative of their overall metabolism in the body, especially in CD patients, making our findings and, above all, their clinical implications extremely difficult to interpret. In addition, the study design adopted (cross-sectional), the lack of a matched control group and the use of CDAI for defining disease activity, albeit conventionally accepted, may have prevented us from detecting further or more relevant differences among serum AAs concentrations. Eventually, the use of the 3-day food records for estimating protein intake may be regarded as a further study limitation. Nevertheless, the strengths of this exploratory analysis are the use of a large sample size and, also, the first attempt to investigate the relationship between AA profiles, protein intake and inflammatory markers in patients with CD.

## 5. Conclusion

In conclusion, the present analysis showed differences in specific AA concentrations between clinically active and quiescent CD patients as well as according to different protein intakes, especially for some EAAs. Glu and Asp values were positively correlated to serum IL-1β, suggesting a potential link with disease activity. Conversely, Trp concentration was reduced unrelated to CDAI. In light of those promising results, extensive research is needed to understand the mechanisms underpinning the relationship between AAs, disease activity and protein intake in patients with CD.

## Data availability statement

The raw data supporting the conclusions of this article will be made available by the authors, without undue reservation.

## Ethics statement

The studies involving humans were approved by the Ethical Committee of Federico II University Hospital. The studies were conducted in accordance with the local legislation and institutional requirements. The participants provided their written informed consent to participate in this study.

## Author contributions

IC and LS contributed to conception and design of the study. NI, AT, OV, LS, IC, and FC collected the data. IC organized the database, performed the statistical analysis, and wrote the first draft of the manuscript. LS, AT, NI and MF wrote sections of the manuscript. All authors contributed to manuscript revision, read, and approved the submitted version.
