# Supplementary material for: Amino acid profiles, disease activity, and protein intake in adult patients with Crohn’s disease
Source: Front Nutr. 2023 Oct 3;10:1245574. doi: 10.3389/fnut.2023.1245574 (PMC10579601; doi:10.3389/fnut.2023.1245574)
Supplement: Supplementary file 1 [file Data_Sheet_1.docx]

**Table S1. Serum amino acids concentration in patients with CD according to surgery**

|  | **Surgery**  **N=54** | **No Surgery**  **N=49** |
| --- | --- | --- |
| **EAA (µM/L)** |  |  |
| **Valine** | 223 (84.5) | 255 (80.5) § |
| **Leucine** | 133 (61) | 143 (50.5) |
| **Isoleucine** | 71 (26.5) | 78 (25) |
| **Threonine** | 136 (70.5) | 150 (39.5) |
| **Lysine** | 172 (56) | 178 (65.5) |
| **Methionine** | 27 (12) | 28 (8) |
| **Histidine** | 88 (20.5) | 84 (22) |
| **Tryptophan** | 7 (4) | 6 (1.5) |
| **Phenylalanine** | 62 (20) | 64 (16.5) |
| **BCAA** | 428 (172) | 471 (134) |
| **Total EAAs** | 862 (271.5) | 938 (267) |
| **NEAA (µM/L)** |  |  |
| **Glutamic Acid** | 81 (60.5) | 70 (39.5) |
| **Asparagine** | 59 (19) | 61 (14) |
| **Arginine** | 98 (54) | 103 (27) |
| **Aspartic Acid** | 26.5 (16.5) | 23 (12.5)§ |
| **Glutamine** | 660 (164) | 669 (142) |
| **Serine** | 141 (43.5) | 152 (39) |
| **Glycine** | 292 (115) | 272 (103) |
| **Tyrosine** | 61.5 (18.8) | 64 (19) |
| **Alanine** | 384 (136) | 374 (122.5) |
| **Cysteine** | 29 (10) | 31 (13.5) |
| **Total NEAAs** | 1877 (432) | 1842 (420) |
| **Other metabolites (µM/L)** | |  |
| **Ornithine** | 91 (44) | 94 (40) |
| **Citrulline** | 31 (11.5) | 34 (12.5) |
| **Taurine** | 148.5 (72) | 160 (68) |

Data are expressed as median and interquartile range, § p<0.07

**Table S2. Serum amino acids concentration according to protein requirements and separated by disease activity in CD patients**

|  | **Active** | | **Quiescent** | |
| --- | --- | --- | --- | --- |
| **EAA (µM/L)** | **UPI** | **MPI** | **UPI** | **MPI** |
|  | N=28 | N=20 | N=19 | N=36 |
| Valine | 220 (99) | 224 (54) | 229 (91) | 273 (74) |
| Leucine | 127 (47) | 126 (61) | 143 (66) | 151 (50) |
| Isoleucine | 70 (26) | 76 (23) | 74 (26) | 75 (31) |
| Threonine | 134 (69) | 164 (31) * | 138 (60) | 152 (62) |
| Lysine | 170 (38) | 165 (68) | 178 (57) | 195 (55) * |
| Methionine | 26 (7) | 27 (12) | 29 (11) | 29 (9) |
| Histidine | 88 (24) | 78 (25) | 88 (25) | 91 (17) |
| Tryptophan | 6 (2) | 6 (5) | 7 (2) | 7 (4) |
| Phenylalanine | 60 (12) | (60 (30) | 66 (19) | 67 (16) |
| **BCAA** | 415 (164) | 427 (143) | 445 (179) | 500 (151) |
| **Total EAAs** | 825 (247) | 874 (237) | 908 (259) | 1015 (256) |
| **NEAA (µM/L)** |  |  |  |  |
| Glutamic Acid | 86 (42) | 86 (60) | 70 (45) | 61 (52) |
| Asparagine | 59 (16) | 62 (21) | 59 (15) | 60 (17) |
| Arginine | 89 (45) | 101 (40) * | 106 (22) | 110 (38) |
| Aspartic Acid | 24 (15) | 28 (19) | 22 (15) | 23 (15) |
| Glutamine | 669 (175) | 640 (174) | 684 (152) | 697 (138) |
| Serine | 148 (47) | 145 (34) | 145 (48) | 152 (48) |
| Glycine | 288 (156) | 301 (102) | 271 (104) | 282 (82) |
| Tyrosine | 59 (20) | 64 (33) | 61 (21) | 62 (17) |
| Alanine | 380 (155) | 356 (131) | 411 (127) | 372 (129) |
| Cysteine | 31 (14) | 30 (10) | 32 (12) | 29 (10) |
| **Total NEAAs** | 1873 (535) | 1826 (272) | 1900 (509) | 1861 (280) |
| **Other metabolites (µM/L)** | |  |  |  |
| Ornithine | 103 (42) | 93 (52) | 82 (44) | 94 (43) |
| Citrulline | 34 (11) | 32 (12) | 33 (16) | 34 (12) |
| Taurine | 149 (68) | 160 (81) | 159 (102) | 150 (73) |

Data are expressed as median and interquartile range. BCAA (branched-chain amino acid) EAAs (essential amino acids); MPI (Met protein intake); NEAAs (non-essential amino acids); UPI (Unmet protein intake). * p<0.05
